# Huge Inverse Magnetization Generated by Faraday Induction in Nano-Sized Au@Ni Core@Shell Nanoparticles

**DOI:** 10.3390/ijms160920139

**Published:** 2015-08-25

**Authors:** Chen-Chen Kuo, Chi-Yen Li, Chi-Hung Lee, Hsiao-Chi Li, Wen-Hsien Li

**Affiliations:** Department of Physics, National Central University, Jhongli 32001, Taiwan; E-Mails: s751734@hotmail.com (C.-C.K.); chiyenli@alumni.ncu.edu.tw (C.-Y.L.); 992402003@cc.ncu.edu.tw (C.-H.L.); lc205063719710096@rocketmail.com (H.-C.L.)

**Keywords:** amorphous Ni nanoparticle, core/shell structure, faraday induction

## Abstract

We report on the design and observation of huge inverse magnetizations pointing in the direction opposite to the applied magnetic field, induced in nano-sized amorphous Ni shells deposited on crystalline Au nanoparticles by turning the applied magnetic field off. The magnitude of the induced inverse magnetization is very sensitive to the field reduction rate as well as to the thermal and field processes before turning the magnetic field off, and can be as high as 54% of the magnetization prior to cutting off the applied magnetic field. Memory effect of the induced inverse magnetization is clearly revealed in the relaxation measurements. The relaxation of the inverse magnetization can be described by an exponential decay profile, with a critical exponent that can be effectively tuned by the wait time right after reaching the designated temperature and before the applied magnetic field is turned off. The key to these effects is to have the induced eddy current running beneath the amorphous Ni shells through Faraday induction.

## 1. Introduction

It is known that a time-varying magnetic field can be used to generate an electric current in conducting materials. The induced current, known as the eddy current, is driven by the electric field created through Faraday induction by the changing magnetic field [1,2]. As stated in Lenz’s law, the magnetic field of the induced eddy current is opposite to the change in the external magnetic field. The generation of such eddy currents in conducting materials is technologically important and has useful practical applications. For example, it has been widely used to produce electricity by rotating metal coils in a steady magnetic field from a permanent magnet or from an electromagnet. Energy generation can be strengthened by using materials or designs that encourage more efficient induction.

Magnetic materials in the form of nano-sized particles or amorphous structures can generate many novel behaviors. For example, memory and aging effects that reflect the characteristics of slow spin dynamics [3,4] have been observed in magnetic nanoparticles (NPs) [5,6,7,8,9,10]. Many applications based on Ni NPs have been proven to be practical, especially in biomolecule separation [11,12]. It has also been demonstrated that the magnetic properties of crystalline core/shell Au@Ni and Ag@Ni NPs can be controlled by tuning the core size and the shell thickness [13]. On the other hand, the magnetic material in the amorphous structure has an extremely low magnetic anisotropy energy, so that the directions of hard and easy magnetizations are largely relaxed. All these properties are technologically valuable for applications as a soft magnetic material and could be used to tune the behavior. Residual magnetization that lasts for a considerable period of time, obtained by relaxing the strength of the applied magnetic field *H*_a_ has been observed in many systems [14,15,16,17]. However, the residual magnetizations are weak, frequently amounting to only a small percentage (<5%) of the original magnetization prior to the *H*_a_ being turned off. In this article, we report on the design and observation of huge inverse magnetizations induced by Faraday induction in 2.2 nm thick amorphous Ni shells deposited on 2.4 nm crystalline Au NPs cores, marked Au@Ni (core@shell). The induced inverse magnetization can be as large as 54% of the magnetization before turning the *H*_a_ off, with a tunable aging relaxation. An eddy current was induced in the crystalline Au NP. Inverse magnetization was obtained by placing the amorphous Ni in such a way that the eddy current circulated beneath the amorphous Ni shell.

## 2. Results and Discussion

### 2.1. Sample Fabrication and Characterization

The Ni@Au NPs used in this study were fabricated employing the gas-condensation method, using a chamber equipped with two decoupled evaporation sources for separate evaporation of Ni or Au. High-purity Au@Ni spheres (~0.3 g each, 99.99% pure and ~2 mm in diameter) were heated separately using a current source of 60/105 A, and were evaporated at a rate of 0.05 Å/s in an Ar atmosphere using a pressure of 2.3 torr. The evaporated particles were collected on a non-magnetic SS316 stainless steel plate placed 20 cm above the evaporation source and maintained at 77 K. After restoration to room temperature, the NPs, which were only loosely attached to the collector, were stripped off. The samples thus obtained were in powdered form and consisted of a macroscopic amount of individual NPs. There were no substrate or capping molecules on the NPs. The resultant powders were no longer gold yellow but dark black, indicating a blue shift in the absorption bands of the powders into the invisible region, as is the case for most metallic NPs. The blue shifting of the absorption bands is known to be a direct result of quantum confinement [18], where spatial restriction to the conduction electrons will give rise to enlargements of band separations [19].

**Figure 1:**
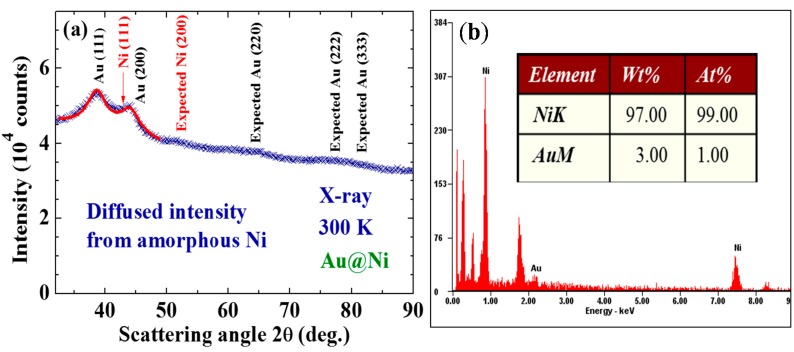
(**a**) X-ray diffraction pattern of the Au@Ni nanoparticles taken at 300 K, revealing a series of diffraction peaks associated with fcc Au together with a strong diffused incoherent intensity distributed over the entire pattern. The arrows indicate the expected peak positions for the fcc Ni. The solid curves indicate the calculated line profiles of the 2.4 nm fcc Au; (**b**) A representative EDXS spectrum, where signals from Au are barely seen but reveal an atomic ratio of Ni:Au = 99:1 for this spatial region of the assembly.

**Figure 2:**
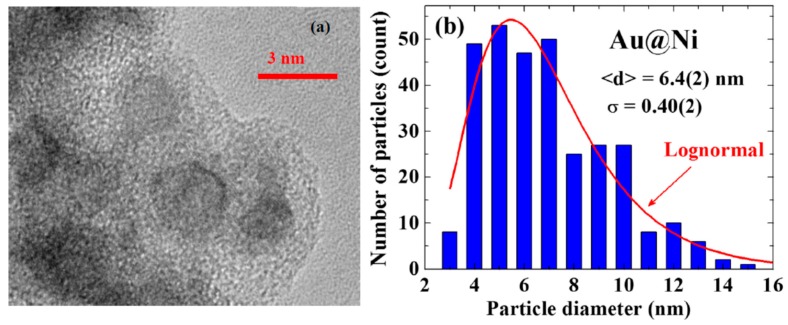
(**a**) Representative TEM images of the Au@Ni NPs, revealing a spherical core/shell structure for the NP; and (**b**) Size distribution obtained from the AFM images. The solid line indicates the results of fits to a lognormal function, giving a mean particle diameter of 6.4 nm with a standard deviation of 0.4.

X-ray diffraction patterns, AFM/SEM/TEM images, and EDXS spectra were all used to characterize the samples. No obvious differences were found in the X-ray diffraction patterns taken from different portions of the sample. The X-ray diffraction pattern for each portion of the sample revealed a series of broad but well-defined diffraction peaks from the crystallized face-centered cubic (fcc) Au together with strong incoherent diffused intensity distributed over the entire pattern, and noticeable but very weak diffraction peaks associated with crystallized fcc Ni (Figure 1a). Elemental analysis using EDXS spectra taken from 12 different portions of the assembly gave an atomic ratio of Ni:Au = 95(2):5(1). A representative EDXS spectrum is shown in Figure 1b. It appears that the strong diffused intensity in the diffraction pattern is mainly from the noncrystalline Ni, and the main component of the NPs is indeed the noncrystalline Ni atoms. No diffraction signals from NiO may be identified in the X-ray diffraction pattern. The TEM images (Figure 2a) show the NPs to have a spherical core/shell structure, with the images of the cores being considerably darker than those of the shells. It is known that the electron density of Au is 1.82 times that of Ni. The NPs thus have a core/shell structure with crystalline Au in the core covered with an amorphous Ni shell. Size analyses based on the AFM images revealed that particle sizes of the NP assemblies can be described using a lognormal distribution, with a mean particle diameter of 6.4(2) nm and a standard deviation of 0.40(2) (Figure 2b). Mean particle diameter of the crystalline Au was determined by fitting the diffraction peaks to the diffraction profiles of finite sized particles, assuming a lognormal size distribution for the NP assembly [20]. The mean particle diameter thus determined for the Au cores is 2.4 nm. Knowing the size of the Au core and the atomic ratio between Ni and Au, we estimate that the Au@Ni NP consists of 2.4 nm fcc Au in the core covered by a 2.2 nm thick amorphous Ni shell, resulting in a mean particle diameter of 6.8 nm for the Au@Ni, which agrees well with that obtained from the AFM images. The physical processes that produced the present core/shell structure are not yet completely realized. However, the chamber pressure employed during the evaporation plays an essential role. An Ar atmosphere of 2.3 torr is known to be suitable for the formation of crystalized Au NPs [20], but a much low chamber pressure is usually needed in the generation of crystalized Ni NPs.

### 2.2. Magnetic Superspins

**Figure 3:**
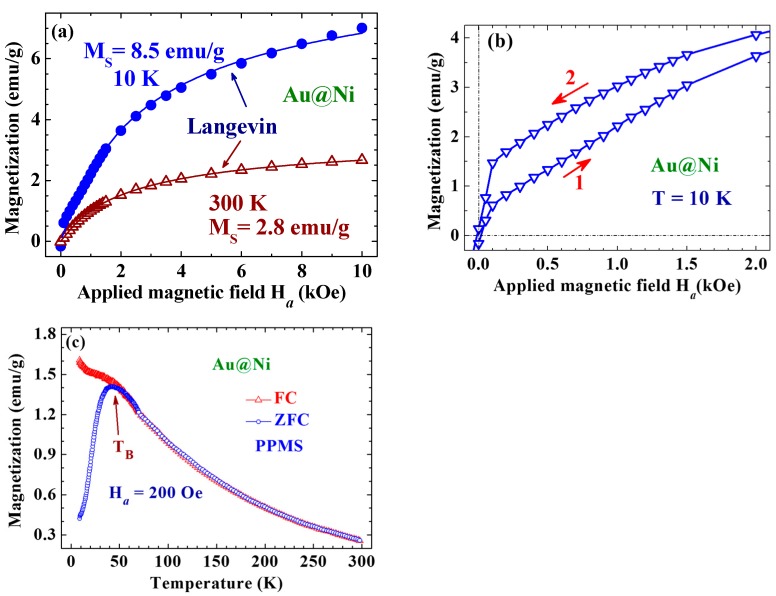
(**a**) *M*(*H*_a_) curves taken at two representative temperatures of 10 and 300 K, where the solid lines indicate the fits of the data to the Langevin profile; (**b**) *M*(*H*_a_) loops taken at 10 K, where the red arrows indicate the directions of the field-changing processes. Magnetic hysteresis with a low coercivity of 40 Oe and a low remanent magnetic moment per unit mass of 0.14 emu/g is revealed; and (**c**) Magnetization as a function of temperature measured in warming with *H*_a_ = 200 Oe, after field-cooling (open triangles) or zero-field-cooling (open circles) from 300 to 2 K. The FC and ZFC curves depart from each other below 50 K, which marks the blocking temperature of the system.

The isothermal magnetization *M*(*H*_a_) curves can be described by a Langevin profile, with the saturation magnetization *M*_S_ reaching 8.5(2) and 2.8(2) emu/g at 10 and 300 K, respectively (Figure 3a). The size polydispersity of the assembly was considered [20] when describing the observed *M*(*H*_a_) curves. The solid curves in Figure 3a indicate the results of the fits for $M(H_{a})=\sum_{i}n_{i}\mu_{pi}\left\{\coth\left(x_{i}\right)-\frac{1}{x_{i}}\right\}$ with $x_{i}\equiv \frac{\mu_{pi}H_{a}}{k_{B}T}$, obtained by knowing the size distribution of the assembly (Figure 2b) and assuming a lognormal type of particle moment dependency on the particle size $\mu_{pi}(d_{i})=\mu_{pm}\exp\left\{-\frac{{\left(\ln d_{i}-\ln d_{c}\right)}^{2}}{2\pi w}\right\}$, with the maximum particle moment μ*_pm_*, the mean particle diameter *d_c_* and width *w* of the moment distribution being the fitting parameters. Eleven *n*_i_’s generated from the size distribution curve (solid curve in Figure 2b) covering *d*_i_ from 1.4 to 11.4 nm were used in the fit. No significant differences in the results from the fit were found when more terms of *n*_i_ were employed. The saturation magnetization thus takes the form of $M_{\text{S}}=\sum_{i}n_{i}\mu_{pi}$. The *M*_S_ for the 10 nm Au@Ni NP is about ~15% that of bulk Ni. Magnetic hysteresis can be clearly seen in the *M*(*H*_a_) curves taken below 50 K, with a coercivity of *H*_C_ = 40 Oe and a low remanent magnetic moment per unit mass of *M*_r_ = 0.14 emu/g at 10 K (Figure 3b). The low *M*_S_, *H*_C_ and *M*_r_ observed for the present assembly reflect the amorphous nature of the Ni in the Au@Ni NPs. It is interesting to note that an asymmetric *M*(*H*_a_) loop with respect to the inversion in the field direction was obtained, where the loop opening shifted by 55 Oe along the positive field direction. This behavior suggests the existence of strong exchange bias in the NPs, which can be driven by the correlations between the superspins in the NPs. This is different from the symmetric *M*(*H*_a_) loop observed in crystalline Ni on Au [13]. The temperature dependencies of the FC and ZFC magnetization taken at *H*_a_ = 200 Oe in warming depart from each other below 50 K, showing a blocking temperature of *T*_B_ = 50 K for the Au@Ni NPs (Figure 3c).

### 2.3. Inverse Faraday Magnetization

**Figure 4:**
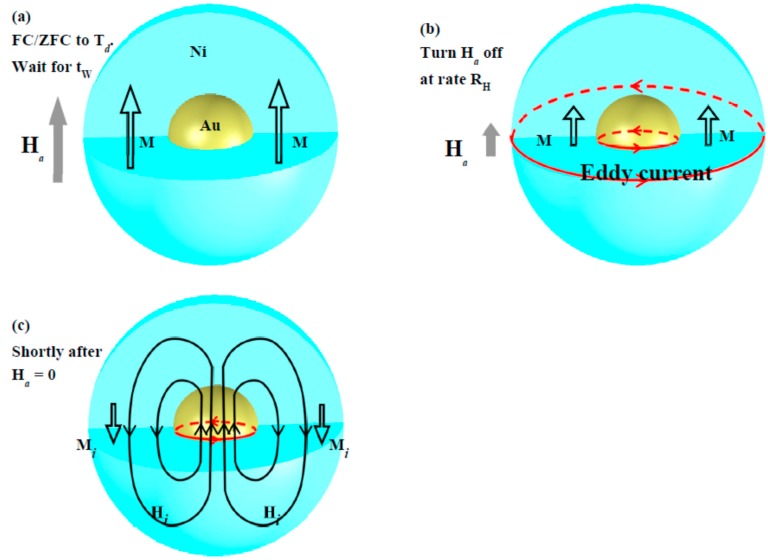
Schematic representations of the applied magnetic field *H*_a_, induced eddy current, induced magnetic field *H*_i_ and magnetization M that developed in the Au@Ni nanoparticle in three representative stages: (**a**) after field-cooling or zero-field-cooling to the designated temperature and after waiting for a designated period of time, where the Ni moments are aligned by the *H*_a_ and giving rise to an M; (**b**) at an intermediate time as *H*_a_ is being cut off, where counterclockwise circulating eddy currents (red circles) have been induced in the Au core and in the Ni shell; and (**c**) shortly after *H*_a_ reaches zero, where the eddy currents in the Ni shell have dissipated while those in the Au core (with a lower resistivity) are still circulating to produce an inverse magnetic field *H*_i_.

Inverse magnetization was generated through the following steps: (1) Field-cooling (FC) at *H*_a_ or zero-field-cooling (ZFC) of the Au@Ni NPs to the designated temperature *T*_d_. If a ZFC process is used, apply *H*_a_ upon reaching *T*_d_, which will allow the development of a net magnetization M in the NP assembly. (2) Remain at *T*_d_ with *H*_a_ for a period of time, which marks the wait time *t*_w_. In the case of NPs with a slow spin dynamical response, the wait time can have a big effect on the net magnetization that develops in the assembly as well as on the resultant relaxation profile (Figure 4a). (3) Turn the *H*_a_ off at a selected field reduction rate *R*_H_ ≡ d*H*_a_/d*t*. An *R*_H_ of −500 or −200 or −100 Oe/s was used in the present study. The induced Faraday electric potential will trigger eddy currents in both the Au core and Ni shell. The changes in flux are considerably larger in the amorphous Ni than in the crystalline Au. Taking the *H*_a_ as pointing in the upward direction, the eddy currents will circulate counterclockwise in the horizontal plane (Figure 4b). (4) The induced current will continue to run for a short period of time after *H*_a_ reaches zero. It appears (see below) that a huge inverse magnetization *M*_i_ opposite to that of the original M could develop in the assembly. The appearance of *M*_i_ can be linked to the eddy current in the Au core, which can be expected to last for a longer time than that in the amorphous Ni, because of the eddy current in the amorphous Ni (with a considerably higher resistivity) will dissipate much faster than that in the crystalline Au [21]. The counterclockwise circulating eddy current in the Au core generates an *H*_i_ opposite to the *H*_a_ on the two sides of the Au NPs, which aligns the neighboring Ni moments downward to produce an inverse magnetization (Figure 4c). The *M*_i_ relaxes gradually with time, with a time constant typically in the range of a few tens of minutes.

**Figure 5:**
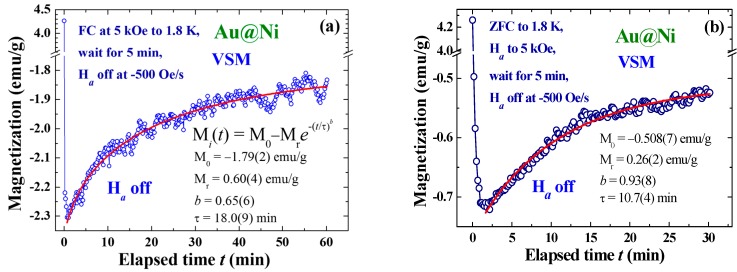
(**a**) Temporal evolution of the induced magnetization recorded after field-cooling at *H*_a_ = 5 kOe from 300 to 1.8 K, followed by a wait time of 5 min before turning the *H*_a_ off at a rate of −500 Oe/s; and (**b**) temporal evolution of the induced magnetization recorded after zero-field-cooling from 300 to 1.8 K, followed by turning the *H*_a_ on to 5 kOe and waiting for 5 min before turning *H*_a_ off at a rate of *R*_H_ = −500 Oe/s.

Inverse magnetization, induced by turning the *H*_a_ off, reaching as high as 54% of the original M (before turning *H*_a_ off) was observed in the FC processes at *H*_a_ = 5 kOe to 1.8 K, with *t*_w_ = 5 min and *R*_H_ = −500 Oe/s (Figure 5a). The induced *M*_i_ persisted for a significantly long period of time, weakening by only 22% one hour after induction. The relaxation of *M*_i_ with time may be described by an stretched exponential decay profile with a dynamic exponent *b* for the temporal parameter: *M*_i_(*t*) = *M*_0_ − *M*_r_exp{(−*t*/τ)*^b^*} (solid curve in Figure 5a) to give *b* = 0.65(6) and τ = 18.0(9) min. *M*_i_(*t*) departs significantly from the exponential decay profile at a high *t* when assuming an exponent of *b* = 1 for the expression. The extremely long relaxation of *M_i_* reflects the existence of strong coupling among the Au@Ni NPs, even though the NPs were only loosely packed. The appearance of a spin-glass type memory effect in the Au@Ni NPs could be due to the spin frustration of the amorphous Ni on the shell [22,23,24]. Although the effects generated in the ZFC processes were smaller they were still clearly visible (Figure 5b), with the induced *M*_i_ reaching 17% with *b* = 0.93(8) and τ = 10.7(4) min for the temporal evolution of the order parameter.

**Figure 6:**
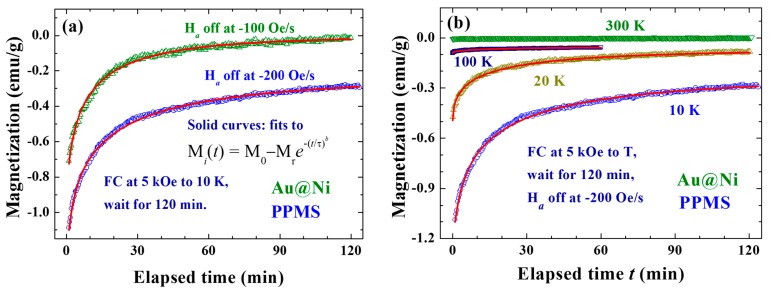
(**a**) Temporal evolution of the magnetization recorded after field-cooling at *H*_a_ = 5 kOe from 300 to 10 K, followed by waiting for 120 min before turning the *H*_a_ off at rates of −200 Oe/s (open circles) and −100 Oe/s (open triangles); and (**b**) temporal evolution of the magnetization recorded after field-cooling at *H*_a_ = 5 kOe from 300 K to four representative temperatures, followed by waiting for 120 min before turning the *H*_a_ off at a rate of −200 Oe/s. The solid lines indicate the fits of the data to the exponential profile listed in (**a**).

Interestingly, the induced *M*_i_ was very sensitive to the field reduction rate *R*_H_. Inverse magnetizations reaching 20% and 12% of the original M were found in the FC process to 10 K at *R*_H_ = −200 and −100 Oe/s, respectively, with *H*_a_ = 5 kOe and *t*_w_ = 120 min (Figure 6a). Compared to the 54% obtained with *R*_H_ = −500 Oe/s (but cooled to 1.8 K), the effect at *R*_H_ = −200 Oe/s is ~2.7 times smaller. Although it can be expected that a higher *R*_H_ will generate a larger eddy current and stronger *H*_i_, the *M*_i_, however, will not monotonically increase with increasing *R*_H_ but will eventually become saturated when all spin domains are aligned accordingly. Note that it cannot be anticipated that *M*_i_ will reach 100% of the original M, since only a portion of the spin domains experiences downward *H*_i_ (Figure 4c). In addition, the induction was also found to be sensitive to temperature. Although it was much reduced at higher temperatures, the induction was still visible even at 300 K. The induced *M*_i_ was reduced to 9, 2.5 and 0.3% of the original M at 20, 100 and 300 K, respectively (Figure 6b). It is possible that the change of resistivity of the Au core plays an important role in the reduction of *M*_i_ at high temperatures. The resistivity ρ of Au increases by a factor of ~100 upon warming from 10 to 298 K [21]. This change will certainly affect the eddy current, hence the magnitude of the Faraday induction. However, the changes in ρ and in *M*_i_ do not linearly correspond. We believed that other mechanisms such as superspin correlation and superspin frustration also play essential roles in current observations. Surprisingly, *M*_i_ relaxed to a sizable value, rather than to zero. The *M*_i_ generated in a FC process at *H*_a_ = 5 kOe to 10 K with *t*_w_ = 120 min, stabilized at *M*_0_ = −0.230(5) and −0.005(3) emu/g when field reduction rates of *R*_H_ = −200 and −100 Oe/s were used, respectively (Figure 6a).

**Figure 7:**
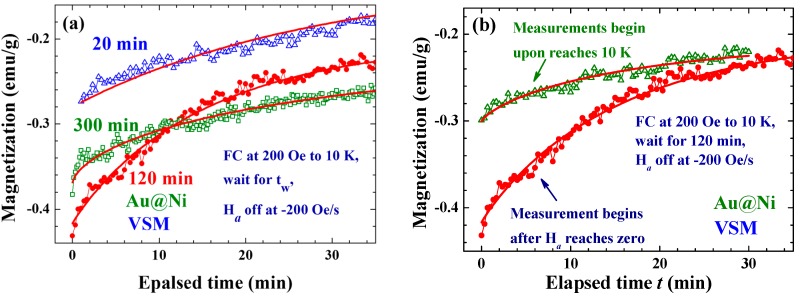
(**a**) Comparison of the temporal evolution of the induced inverse magnetizations after wait times of 20 min (open triangles), 120 min (filled circles) and 300 min (open squares), through field-cooling at *H*_a_ = 200 Oe from 300 to 10 K and a field reduction of *R*_H_ = −200 Oe/s; and (**b**) temporal evolution of the inverse magnetization recorded upon reaching 10 K (open triangles) and when the *H*_a_ is completely off (filled circles), in the process of field-cooling at *H*_a_ = 200 Oe from 300 to 10 K, followed by a wait time of *t*_w_ = 120 min before turning the *H*_a_ off at a rate of *R*_H_ = −200 Oe/s. The solid lines indicate the fits of the data to the exponential profile listed in (a).

Inverse magnetization can also be induced by a weak *H*_a_, reaching 27% of the original M in the FC process to 10 K at *H*_a_ = 200 Oe, with *t*_w_ = 120 min and *R*_H_ = −200 Oe/s (filled circles in Figure 7a). A longer wait time will generate a larger effect, but it appears that there is an upper limitation to the wait time for maximum effect (Figure 7a), longer than which *t*_w_ plays a significantly less essential role. The wait time for maximum induction is likely to be ~120 min for the present Au@Ni NPs assembly. It is surprising to find that the conduction of the magnetization measurement can also affect the strength of the induced *M*_i_, as demonstrated in Figure 7b. The induced *M*_i_ dropped from −0.43 emu/g (*i.e.*, a 27% induction) to −0.30 emu/g, if measurements were made during the waiting period. This interesting behavior reflects again that the Faraday induction of the amorphous Ni in Au@Ni NPs is very sensitive to changes in the external disturbances. In addition, there is essentially no difference found between the *M*_i_ that is induced when using a Vibrating Sample Magnetometer (VSM) or a Physical Property Measurement System (PPMS). We, however, noticed that the *M*_i_(*t*) obtained when using VSM relaxes at a slightly but noticeably slower rate, reflecting that the vibrating-sample used in VSM measurement can also affect *M*_i_. The relaxation parameters obtained from the fits of the *M*_i_(*t*) curves to *M*_0_ − *M*_r_exp{(−*t*/τ)*^b^*} are listed in Table 1. The dynamic exponent *b* reflects the changes of relaxation rate of *M*_i_ with the evolution of time. A value for *b* of smaller than 1 indicates the relaxation is slowing down, whereas a value for *b* of larger than 1 indicates that the relaxation is speeding up. It is interesting to see that the dynamic exponent *b* of the temporal evolution of the order parameter *M*_i_ are all smaller than 1, showing that *M*_i_ relaxes at a significantly slower rate with the evolution of time. It is very unlikely that the slowing down of the *M*_i_ relaxation is linked to the growth of the magnetic correlation length, as is frequently expected, but reflects the longer and longer time it can take for domain superspins to randomly flip. This can be understood by assuming that there are wide dimensions of ferromagnetic spin domains in the amorphous Ni shell of each NP. The mean time between two random flips of the domain superspin, known as the Neel relaxation time, is longer for a larger spin domain. The magnetic relaxation is dominated by larger spin domains at later times, giving rise to the slowing down of the relaxation.

**Table 1:** Parameters obtained from the fits of the *M*_i_(*t*) curves to the expression *M*_i_(*t*) = *M*_0_ − *M*_r_ exp{(−*t*/τ)*^b^*}. The * indicates that the measurements were made during the waiting period.

Process	*H*_a_ (kOe)	*T*_d_ (K)	*R*_H_ (Oe/s)	*t*_w_ (min)	*M*_0_ (emu/g)	*M*_r_ (emu/g)	τ (min)	*b*
FC	5	1.8	−500	5	−1.79(2)	0.60(4)	18.0(9)	0.65(6)
ZFC	5	1.8	−500	5	−0.508(7)	0.26(2)	10.7(4)	0.93(8)
FC	5	10	−100	120	−0.005(3)	1.00(3)	8.1(4)	0.53(2)
FC	5	10	−200	120	−0.230(5)	1.24(3)	10.5(5)	0.45(1)
FC	5	20	−200	120	−0.070(2)	0.424(5)	12.2(2)	0.49(1)
FC	5	100	−200	120	−0.053(1)	0.038(2)	21(2)	0.52(2)
FC	0.2	10	−200	20	−0.126(1)	0.154(2)	29.0(4)	0.99(2)
FC	0.2	10	−200	120	−0.201(7)	0.216(2)	15.7(1)	0.94(1)
FC	0.2	10	−200	300	−0.201(1)	0.168(2)	33.6(3)	0.65(1)
FC *	0.2	10	−200	120	−0.202(1)	0.098(3)	18.2(4)	0.77(3)

### 2.4. Memory Effect

**Figure 8:**
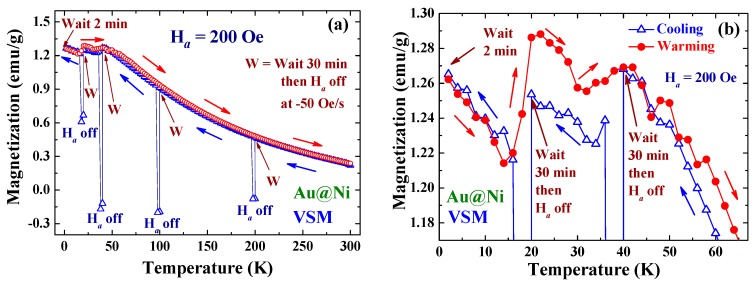
(**a**) Temperature dependencies of the magnetization collected at temperatures measured in steps of 2 K in the processes starting with cooling (open triangles) from 300 K with *H*_a_ = 200 Oe. The W’s at 200, 100, 40 and 20 K mark the process of turning the *H*_a_ off at a rate of *R*_H_ = −50 Oe/s for 3 min, after waiting for 30 min upon reaching that temperature. The arrows indicate the directions of the temperature-changing processes. The open circles indicate the magnetization measured in warming, after waiting for 2 min upon reaching 2 K. (**b**) Low temperature portions of the M(T) curves displayed in (a), showing step-like increases of M around 15 and 35 K, where *H*_a_ has been turned off during cooling. No such behaviors are observed around 100 and 200 K.

A memory effect in the magnetization was revealed below the blocking temperature but not above, as demonstrated in the relaxation measurements shown in Figure 8a,b. These M(T) loops were collected in the processes beginning with the slow cooling from 300 K in *H*_a_ = 200 Oe. Magnetization was recorded in steps of 2 K after the temperature was stabilized. Cooling was temporarily stopped for 30 min upon reaching 200 K, followed by turning the *H*_a_ off at a rate of –50 Oe/s to allow the magnetization to relax downward for 3 min. Cooling was resumed after reapplying *H*_a_ to reach 200 Oe. The same process of temporarily stopping the cooling and turning the *H*_a_ off-and-on was conducted at 100, 40 and 20 K. There was a 2 min wait upon reaching the base temperature of 2 K before warming the sample to 300 K using the same temperature steps. Interestingly, the M(T) curve taken in the warming process displays step-like increases at 15 K (by 6%) and 35 K (by 1%), where *H*_a_ was temporarily terminated in the cooling process (Figure 8b). No abrupt changes of M were observed at 100 and 200 K. Note that the time interval between the cooling and warming processes at 20, 40, 100 and 200 K are 82, 172, 355 and 617 min, respectively. It appears that the memory effect could last for 172 min, as it does appear around 40 K. The disappearance of the memory behavior at 100 and 200 K could be because the time interval has exceeded the time that the memory can last or that the temperatures are well above *T*_B_. The memory effect in nanoparticle systems has been attributed to the existence of significant dipole interactions among particle superspins and/or the broad distribution of relaxation times in the NP assembly [25]. In the present case, the NPs are very loosely packed and the interparticle interaction is insignificant. Size polydispersity of the assembly then plays a major role in the appearance of the memory effect. The anisotropy energy barriers for random flips of domain superspins can generate not only stretched exponential relaxation of the induced magnetization but also give rise to the memory effect.

## 3. Experimental Section

The X-ray diffraction measurements for structural investigation were performed on a Bruker D8 ADVANCE diffractometer (Bruker Corporation, Billerica, MA, USA), employing the standard reflection geometry. Chemical analysis by means of energy dispersive X-ray spectroscopy (EDXS) was also performed to characterize the elemental composition of the sample. The EDXS spectra were taken with a HORIBA EX-220 detector attached to a HITACHI S-4200 scanning electron microscope (SEM) (Hitachi Ltd., Tokyo, Japan), employing the standard setup to analyze 12 portions of the sample. The atomic force microscope (AFM) images of the NPs were taken using a Nanoscope-III (Veeco Instruments Inc., Plainview, NY, USA) operated in the tapping mode, where a noncontact technique, with the cantilever tip vibrating at a large amplitude to avoid trapping, was used to profile the sample surface for size analysis. To reduce aggregation of the NPs, the powder was shaken at 30 Hz for 5 min using a Vortex-Genie Mixer (Scientific Instruments Inc., Ringoes, NJ, USA) before it was packed into a thin nonmagnetic cylindrical holder for magnetization measurement. The holder, manufactured by Quantum Design, produced a smooth temperature curve and a background signal that was ~2% of the signal from the sample. The packing fraction *f* of the NP assembly was ~10%, which indicates the ratio of the mass densities of the assembly to that of its bulk counterpart. Note that when the NPs are naturally assembled together, the packing fraction of the resultant powder is low. This is understood to be because the assembly consists of very many loosely connected NP aggregates and the NPs within each aggregate are only weakly linked together, so that the spatial filling factor of the NPs and their aggregates is considerably low when they are naturally packed. The magnetization measurements were performed, employing the standard setups, on a Physical Property Measurement System or on a Vibrating Sample Magnetometer, both manufactured by Quantum Design (San Diego, CA, USA).

## 4. Conclusions

This structure of a nano-sized amorphous Ni shell deposited on a crystalline Au nanoparticle in the core can further be developed into a nano-magnetic switch, when is using the on-or-off of an external magnetic field to change the magnetic polarization of the structure that switching the connection from one loop to the other. Fabrication of the structure into nano-leads to extract the eddy current in the core can provide a power source for a nano-device, operated by a swinging magnet or by turning an external magnetic field on-and-off. The key components for inducing inverse magnetization include: (1) The use of a magnetic material in its amorphous form to relax the hard magnetic axis for easy moment alignment and less magnetic anisotropic loss; (2) a conducting material with low resistivity that will support an induction current flowing beneath the magnetic material; and (3) a nano-sized structure to provide more individual induction currents for enhancement of the effect. The small remanence together with low coercivity for the present Au@Ni nanoparticles, show that the magnetic anisotropy energies associated with the superspins in the magnetic domains are low and the magnetic axis is soft. These characteristics are essential for large induction of inverse magnetization. On the other hand, the extremely long relaxation time reflects the appearance of strong inter-domain magnetic interaction for slow spin dynamics. The behaviors observed in the present study are obtained in a very loosely packed Au@Ni nanoparticle assembly, where interparticle interaction was not significant. However, it is expected that the memory effect will be strongly affected by interparticle interaction. It would be interesting to examine the roles played by interparticle interaction on inducing inverse magnetization.
